# Protease-processed amnion: biological rationale and translational constraints

**DOI:** 10.2340/biid.v13.46528

**Published:** 2026-07-22

**Authors:** Raghuram Manickam, Vijaya Nirmala Subramani

**Affiliations:** Department of Oral Pathology, Sri Ramachandra Dental College and Hospital, Sri Ramachandra Institute of Higher Education and Research, Chennai, Tamil Nadu, India

**Keywords:** Amnion membrane, dental regeneration, wound healing, protease-processed amnion, biomaterials, oral surgery

## Abstract

Amnion-derived biomaterials have attracted interest in dentistry because of their anti-inflammatory, anti-scarring, and wound-healing properties. Among available preparation strategies, protease-processed amnion has been proposed as a method to better preserve structural integrity and support cellular responses, with preliminary evidence suggesting accelerated wound healing. This mini review examines the biological rationale for protease-processed amnion, compares it with conventional membrane processing approaches, and evaluates its possible role in extraction socket healing, periodontal regeneration, and oroantral repair. Available evidence suggests promising preclinical performance, but clinical support in intraoral settings remains limited. Key barriers include variability in processing, sterilization, storage, regulatory oversight, and the need for well-designed clinical trials before routine dental use can be recommended.

**KEY MESSAGES:**

- Protease-processed amnion may enhance preservation of amnion structural and bioactive components compared with conventional processing methods.
- Preclinical studies suggest potential improvements in cellular response and wound healing, although evidence remains limited and not validated in intraoral models.
- Clinical translation is limited by a lack of intraoral randomized controlled trials and standardized outcome measures.

## Introduction

Amniotic membrane has a long history as a biologically active tissue scaffold in regenerative medicine, and recent dental literature has expanded its use in oral and maxillofacial surgery, periodontics, endodontics, and guided bone regeneration [1, 2]. Its appeal lies in a combination of low immunogenicity, anti-inflammatory activity, and a native extracellular matrix that can support healing [1, 3]. However, the term ‘amnion biomaterial’ covers several distinct products prepared by different methods, and these processing differences are important because they influence structural integrity, bioactivity, handling, and clinical performance [3].

This mini review focuses specifically on protease-processed amnion as the emerging preparation strategy of interest, using Paulraj et al. as the principal example because the original manuscript places substantial emphasis on this method [4]. At present, available literature on protease-processed amnion in dental or intraoral applications is extremely limited, and Paulraj et al. represents the most detailed experimental contribution within this emerging processing class rather than a large established body of evidence. To maintain a balanced perspective, the discussion also considers other amnion approaches and compares them with established dental biomaterials. The aim is to critically assess whether this preparation strategy offers a meaningful translational advantage worth further investigation.

This article is structured as a focused narrative mini-review examining a single emerging processing strategy within the broader context of amnion-based biomaterials in dentistry.

### Processing methods

Conventional amnion products are commonly prepared by dehydration, freeze-drying, decellularization, or irradiation-based sterilization, each of which can alter matrix ultrastructure and may reduce retention of native proteins and growth factors [3]. In contrast, protease-mediated recovery has been presented as a gentler method intended to preserve the collagen scaffold and associated signaling molecules more effectively [4]. The theoretical advantage is that if the membrane retains more of its native architecture, it may better support cell migration, adhesion, epithelialization, and soft-tissue repair [3, 4].

That said, preservation of structure alone does not guarantee superior dental performance. The oral cavity imposes mechanical loading, salivary contamination, and a dense microbial environment that may affect degradation, fixation, and healing behavior in ways that are not captured in extraoral wound models [1, 3]. For that reason, any claim of superiority must be interpreted cautiously until validated in site-specific oral studies [4].

### Biological rationale

The appeal of amnion-based membranes in dentistry is rooted in their extracellular matrix composition and bioactive signaling profile. Reviews of amniotic membranes note potential anti-inflammatory, anti-scarring, and pro-healing effects, with reported utility in oral soft tissue repair, vestibuloplasty, recession coverage, and guided tissue regeneration [1, 2]. These benefits provide a plausible biological basis for their use as a barrier membrane and wound dressings in selected dental procedures.

Protease-processed amnion may strengthen this biological rationale by avoiding harsh processing steps that can denature structural proteins or reduce retention of endogenous mediators [4]. This may include improved preservation of Extracellular matrix (ECM)-bound signaling molecules such as Vascular Endothelial Growth Factor (VEGF) and Transforming Growth Factor Beta (TGF-β), as well as reduced degradation of laminin and fibronectin, which are critical for cell adhesion and early wound matrix organization. If confirmed, this could be clinically relevant in procedures where rapid epithelial coverage and stable soft tissue healing are priorities, such as extraction sockets and oroantral communications [1]. However, direct quantitative evidence demonstrating superior retention of these molecules following protease-processed amnion in intraoral-relevant conditions remains limited, as the current evidence base is still dominated by laboratory and non-oral data [4].

### Dental applications

#### Extraction socket healing

Extraction sockets are biologically active wounds that undergo a coordinated sequence of hemostasis, inflammation, proliferation, and remodeling [5]. Amniotic membrane may be useful as a socket dressing because it can promote early epithelial coverage and support soft tissue stabilization, which could be advantageous in difficult healing situations [2, 6]. The available clinical literature, however, is still limited and heterogeneous, so protease-processed amnion should be regarded as an investigational adjunct rather than a proven replacement for established socket management approaches [4].

#### Periodontal regeneration and guided tissue regeneration

The use of amnion as a Guided Tissue Regeneration membrane has been reported in periodontics, including recession defects and periodontal wound coverage [1, 2]. In theory, a protease-processed membrane with improved matrix preservation could function both as a barrier and as a biologically active scaffold [4]. Even so, traditional collagen membranes remain the most established comparators in this setting, and the review should acknowledge that the evidence supporting standard materials is much stronger than the evidence supporting any new amnion preparation method [7].

#### Oroantral repair and oral soft tissue closure

Amniotic membrane has also been used for oral wound closure, including repair of oroantral communications, with reports of favorable epithelialization and healing [8]. These applications support the concept that the membrane can function as a biologically active coverage material in oral and maxillofacial surgery [1]. For protease-processed amnion, the translational question is whether enhanced structural preservation meaningfully improves performance beyond what has already been reported for other amnion preparations.

### Critical comparison with other biomaterials

Conventional collagen membranes, xenogeneic membranes, synthetic barrier membranes, and connective tissue grafts have known handling characteristics, regulatory pathways, and evidence bases in clinical dentistry. At present, protease-processed amnion lacks head-to-head randomized clinical trials against collagen-based barrier membranes, which remain the gold standard in guided tissue regeneration. By comparison, protease-processed amnion currently has a narrower evidence base and lacks standardized intraoral outcome data, which limits direct comparison [2, 3].

The central scientific issue is therefore not whether amnion can support healing in principle, but whether this specific processing method offers a reproducible and clinically meaningful advantage over existing alternatives. Without head-to-head studies, claims of superiority should be softened to reflect hypothesis-generating rather than practice-changing evidence.

### Translational limitations

Several barriers must be addressed before clinical adoption can be considered. These include manufacturing consistency, sterilization assurance, storage stability, batch-to-batch reproducibility, cost effectiveness, and regulatory classification. In dentistry, there is an additional challenge: intraoral biomaterials must withstand saliva, bacterial challenge, and constant mechanical stress while maintaining predictable degradation and fixation.

Clinical translation will require well-designed randomized controlled trials, standardized outcome measures, and comparisons against accepted membranes and soft tissue grafting methods. At present, the evidence is sufficient to justify further study but not enough to recommend routine clinical use.

## Conclusion

Protease-processed amnion processing is an interesting and potentially useful refinement in the preparation of biologically active membranes for wound healing. In dentistry, it may have future value for extraction socket management, periodontal regeneration, and oroantral repair, but current support is still largely preclinical or based on limited clinical reports. At present, the available evidence supports further investigation rather than routine clinical adoption, and future studies should prioritize direct comparison with established dental biomaterials under intraoral conditions.

## Data Availability

Data sharing is not applicable to this article as no new data were created or analyzed in this study.
